# Observations of Immuno-Gold Conjugates on Influenza Viruses Using Waveguide-Mode Sensors

**DOI:** 10.1371/journal.pone.0069121

**Published:** 2013-07-11

**Authors:** Subash C. B. Gopinath, Koichi Awazu, Makoto Fujimaki, Kazufumi Shimizu, Takayuki Shima

**Affiliations:** 1 Electronics and Photonics Research Institute, National Institute of Advanced Industrial Science and Technology, Tsukuba, Ibaraki, Japan; 2 Open Research Center for Genome and Infectious Disease Control, Nihon University School of Medicine, Itabashi-ku, Tokyo, Japan; University of North Carolina Greensboro, United States of America

## Abstract

Gold nanoparticles were conjugated to an antibody (immuno-AuNP) against A/Udorn/307/1972 (H3N2) influenza virus to detect viruses on a sensing plate designed for an evanescent field-coupled waveguide-mode sensor. Experiments were conducted using human influenza A/H3N2 strains, and immuno-AuNP could detect 8×10^5^ PFU/ml (40 pg/µl) intact A/Udorn/307/1972 and 120 pg/µl A/Brisbane/10/2007. Furthermore, increased signal magnitude was achieved in the presence of non-ionic detergent, as the virtual detection level was increased to 8×10^4^ PFU/ml A/Udorn/307/1972. Immuno-AuNPs were then complexed with viruses to permit direct observation, and they formed a ring of confined nanodots on the membrane of both intact and detergent-treated viruses as directly visualized by scanning electron microscopy. With this complex the detection limit was improved further to 8×10^3^ PFU/ml on anti-rabbit IgG immobilized sensing plate. These strategies introduce methods for observing trapped intact viruses on the sensing plates generated for optical systems.

## Introduction

Influenza viral infection is a commonly occurring disease all over the world, and when avian and human influenza viruses simultaneously infect intermediate hosts, novel virus occurs because of the genetic reassortment [1]–[3]. Emerging or re-emerging virulent influenza strains can cause infections of epidemic proportions and severely affect human and animal populations [4]–[7]. A classic example of newly emerging strains is the recently emerged H1N1 viral strain (A/California/04/2009), which was implicated in the 2009 flu pandemic among humans and is known as “swine flu”. The World Health Organization named this pandemic strain as A(H1N1)pdm09. Recent evidence indicates that a new strain of influenza A (H3N2)v (v stands for variant) has the gene encoding the matrix protein from the influenza A (H1N1)pdm09 virus. In addition, a gene encoding hemagglutinin (HA) of (H3N2)v is related to the strain found circulating among individuals with chronic health issues in the 1990s [8]. Currently, among several types of influenza viruses classified based on 16 HA and 9 Neuraminidase, subtypes H3N2 and H1N1 are circulating in humans [9]. In addition, a new HA was found to occur in a distinct lineage of influenza A virus in little yellow-shouldered bats and was designated as H17 [10]. A H3 HA gene from an avian source was introduced to human H2N2 influenza virus, and it caused severe pandemics in the year 1968 [11]. The emergence of new viruses poses problems with regard to economic impact, clinical surveillance, and control measures [7], and thus, a system is required for earlier detection of influenza viruses. Early diagnosis is considered as one of the key issues to prevent the further spread of viruses and facilitate influenza therapy [12]. HA is the major determinant of influenza variants and is a major homo-trimeric protein on the membrane of influenza viruses that is involved in membrane fusion with the host cell during infection [13]–[15].

At present, several anti-HA detection systems use anti-HA probes, including anti-influenza aptamers and antibodies, to detect viruses [16]–[19]. Several of these diagnostic methods have been shown to be capable of detecting and characterizing influenza viruses [18], [20]–[25]. Immunochromatography, real-time reverse transcription polymerase chain reaction and other sensor-based techniques are presently in use for the identification of influenza viruses and for discrimination between influenza A and B. In the present study, we have formulated an alternative approach with an evanescent field-coupled waveguide-mode (EFC-WM) biosensor [26]; this type of sensor has been used to detect biomolecular interactions with high sensitivity [18]–[20], [27]–[34]. Previously, using an antibody against HA, we developed a method based on this type of sensor for detecting HA in viruses that infect humans or birds [18], [19]. In the present study, to enhance the spectral signal from the waveguide sensor, we used gold nanoparticles (AuNP), which are considered to be an attractive tool for bio-nanosensor development and absorb visible light at approximately 520 nm because of excitation of plasmons [35], [36]. For influenza detection, we used an AuNP-conjugated anti-A/Udorn/307/1972 antibody together with a silicon-based sensing plate operating in a waveguide mode to detect the H3N2 influenza strains (A/Udorn/307/1972 and A/Brisbane/10/2007).

## Results and Discussion

Different sensing systems were previously proposed to detect and discriminate influenza viruses in both human and bird samples with varying detection limits [17], [20]–[25]. In general, sensors are expected to have portability, sensitivity, selectivity, simplicity, reliability, precision, and stability. To achieve these characteristics, in the present study, interactive analyses were conducted on the sensing plate using the waveguide sensor, where the affinity of an antibody targeting A/Udorn/307/1972 was evaluated for H3N2 strains. To observe these strains on the sensing plate, the antibody was conjugated with different sizes of AuNPs. This type of AuNP is commonly used in sensor development and has unique characteristics, such as ease of dispersal in the water, compatibility with surface functionalization for conjugation of biomolecules, and capability to be tailored to desired nanosizes [34], [35], [37], [38].

### Preparation of AuNPs and Antibody Conjugates

**Figure 1:**
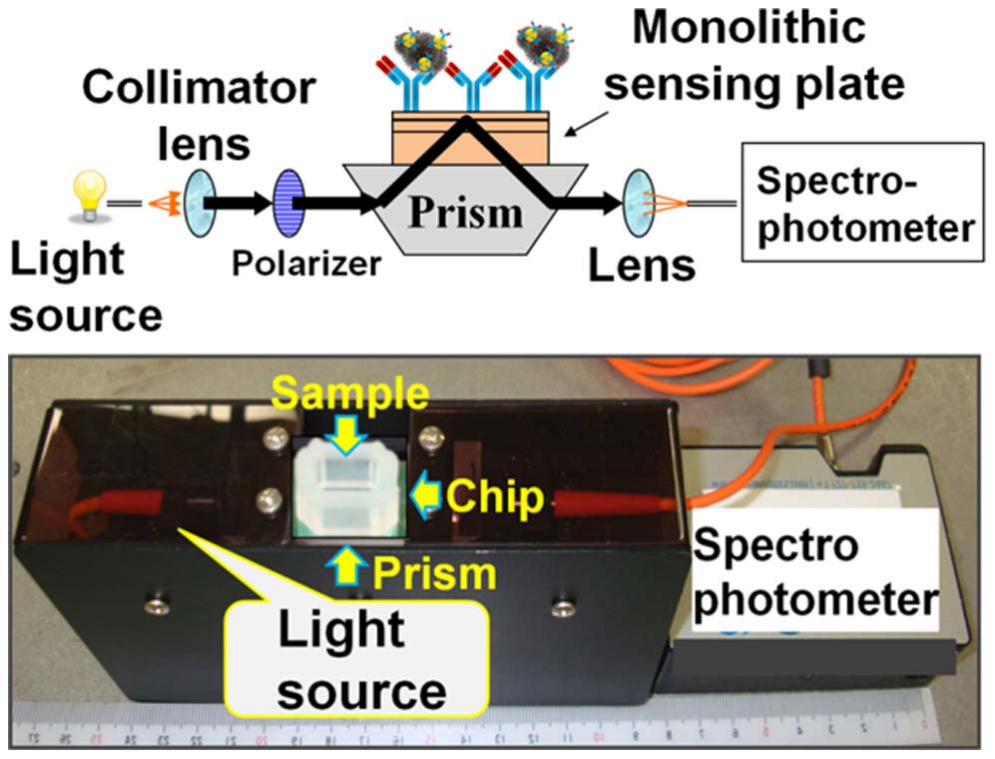
Diagrammatic representations of compact waveguide-mode sensor system. Photograph of the handy portable prototype of the sensing system. The spectral readout system is employed in the setup. Xe lamp was used as the light source.

**Figure 2:**
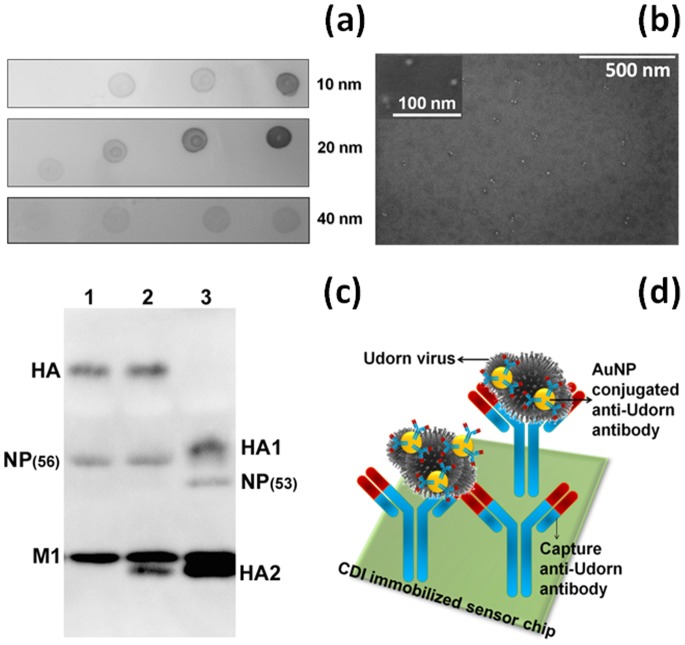
Preliminary analyses with antibody and immuno-AuNPs. (a) Dot-blot analyses with immuno-AuNPs. Different concentrations of immuno-AuNPs were spotted on the nitrocellulose membrane. AuNPs with a diameter of 10, 20, or 40 nm were tested. (b) SEM images for the attachment of immuno-AuNPs on the CDI surfaces. The scale bar is shown. Figure inset for enlarged view. (c) Western blot analyses using the antibody generated for A/Udorn/307/1972. Lane 1: A/Udorn/307/1972 virus with uncleaved HA; Lane 2: virus with partially cleaved HA by 0.5 µg/ml TPCK-trypsin treatment at 34°C for 20 min; Lane 3: virus with completely cleaved HA by 2.5 µg/ml TPCK-trypsin treatment at 34°C for 20 min. The hemagglutinin, HA (72 kDa) is cleaved into HA1 (55 kDa) and HA2 (25 kDa). The nucleocapsid protein, NP (56 kDa) is cleaved into a 53-kDa protein. Virus proteins were separated by 18% SDS-PAGE containing 3 M urea. The anti-Udorn antibody strongly react with HA2 and M1, modestly with HA and HA1, and weakly with NP(56) and NP(53). (d) Representation of the attachment of molecules on the sensing plate. The capture antibody was attached on the *N*,*N*′-carbonyldiimidazole (CDI) surface, followed by the virus and immuno-AuNPs.

To observe the binding affinity between influenza viruses and antibody-conjugated gold nanoparticles (immune-AuNP) on an evanescent field-coupled waveguide-mode (EFC-WM) sensor (Figure 1), we initially prepared 3 sizes of AuNPs with diameters of 10, 20, and 40 nm. The antibody produced against intact A/Udorn/307/1972 in immunized rabbits was conjugated with all 3 different sizes of AuNPs by using surface functionalization chemistry. To ensure the proper attachment of the antibody on the AuNPs, we performed dot-blot analyses on the nitrocellulose membrane. The specific attachment of the antibody on AuNPs was evaluated by using the anti-rabbit IgG. It was confirmed that the AuNPs were properly conjugated with our desired antibody (anti-A/Udorn/307/1972 antibody), and an increase in signal intensity was observed with an increased amount of spotted AuNP-antibody complexes (Figure 2a). Furthermore, these immuno-AuNPs were also immobilized on a sensing plate functionalized with *N,N*′-carbonyldiimidazole (CDI), which can capture proteins. After attachment of the complexes, we observed the surface of the sensing plate under scanning electron microscopy (SEM). The SEM images clearly indicate the chemical attachment of immuno-AuNPs on the CDI-modified surface and with the proper distribution (Figure 2b). To distinguish the physical attachment of all 3 AuNP sizes on the sensing plate in the absence of virus, we attached the AuNP–antibody complexes with different dilutions on surfaces that had been functionalized with the capture antibody and blocked by ethanolamine. Among the 3 sizes (10, 20, and 40 nm) tested, the AuNP-antibody complexes with diameters of 20 and 40 nm underwent physical adsorption that we considered to be non-specific binding. However, the 10-nm immuno-AuNP was completely free from physical attachment, and we therefore concluded that AuNP particles with a diameter of 10 nm can undergo specific interactions with the viral particle (Figure S1). Using these different sizes of AuNP, we established a sensing system for 2 H3N2 strains: virus prepared using Madin-Darby canine kidney (MDCK) cells (A/Udorn/307/1972) and the commercially available A/Brisbane/10/2007 virus.

### Detecting A/Udorn/307/1972 Intact Virus on the Sensing Plate

**Figure 3:**
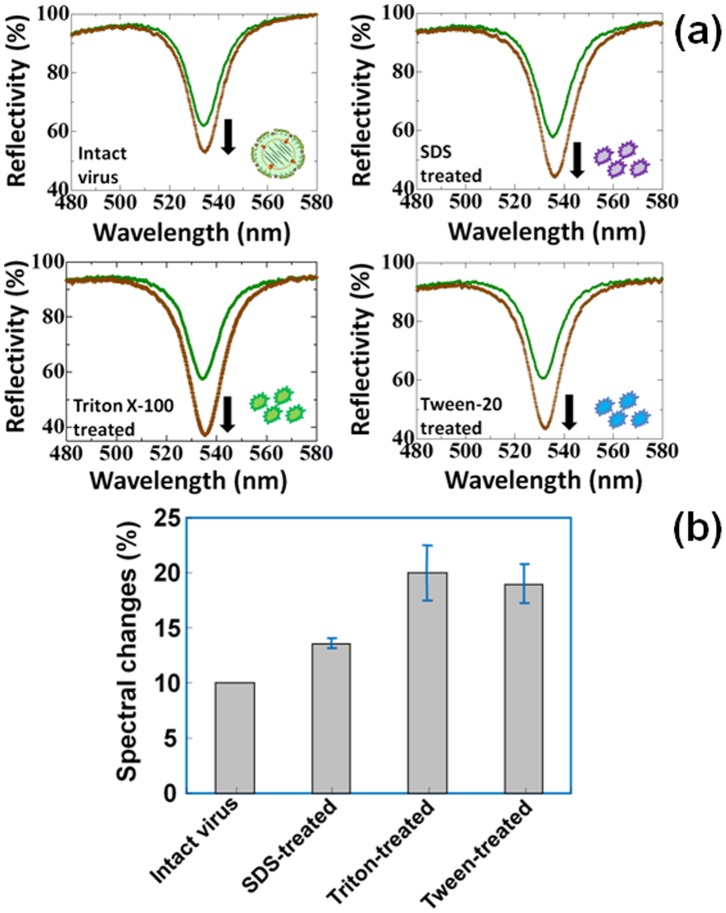
Spectrum shows the interactions of A/Udorn/307/1972 and immuno-AuNP. (a) Intact viruses and those treated with different detergents are shown. The detergents were used at a concentration of 0.5%. Vertical arrows on the figures indicate the direction of the spectral changes. Shift only occurs after the final. The green and brown lines represent the reflectivity measured after the attachment of virus and immuno-AuNPs, respectively, on the CDI surface immobilized with the capture antibody and ethanolamine. (b) Graphical representation of intact and detergent-treated viruses. Error bars are shown with averaged values.

In the waveguide-mode sensing system, initially we conducted experiments with A/Udorn/307/1972 virus. To confirm the binding affinity, we performed western blot analyses, by blotting separated virus proteins on polyvinyl difluoride membrane followed by the attachment of primary and secondary antibodies. As predicted, the antibody used in this study could bind to A/Udorn/307/1972 and the appearance of Hemagglutinin 1 and 2 (HA1 and HA2) were noticed with digestions of virus with trypsin (Figure 2c). To mimic the interactions of the antibody-immobilized AuNPs with viruses, the CDI immobilized surface was functionalized with the capture antibody (anti-A/Udorn/307/1972) but without AuNP-conjugation and then immobilized with the intact test virus. On the sensor chip surface to which the test virus was attached, the antibody-conjugated AuNP (10 nm) was incubated with a dilution of 1∶10 for the measurements in the following sections; this set-up results in a sandwich pattern with the virus (Figure 2d). We initially performed interactive analyses for A/Udorn/307/1972 against immuno-AuNPs, 10% reflectivity was easily achieved with a virus concentration of 8×10^5^ PFU/ml (Figure 3a). We also performed the experiments with 8×10^5^ PFU/ml A/Udorn/307/1972 on a sensing plate immobilized with pre-immune serum obtained from the same rabbit used to develop the anti-A/Udorn/307/1972 antibody to evaluate non-specific reactions of components from the pre-immune serum. These results clearly indicate a lack of cross interactions between the virus and impurities in the serum (Figure S2).

### Effects of Ionic and Non-ionic Detergents on Influenza Detection

We used our sensor to determine the effect of ionic and non-ionic detergents on the strain A/Udorn/307/1972. For these analyses, we used sodium dodecyl sulfate (SDS) as an ionic detergent and Triton X-100 and Tween-20 as non-ionic detergents to dissociate viral proteins. Using the detergents at a concentration of 0.5%, independent experiments were performed as described above. To prevent the complete disintegration of proteins from the viruses by detergent activity, the detergents were used just prior to the experiment and incubated for 20 min in the shell used for the waveguide sensor. In the preliminary experiment with no detergent using intact virus at a concentration of 8×10^5^ PFU/ml on the waveguide sensor, the spectral shift was 10%, measured in terms of reflectivity changes. In contrast, when we treated the same concentration of viruses (8×10^5^ PFU/ml) with the different detergents, the changes of the spectrum were 13.6±0.5, 20±2.5, and 19±1.75 for viruses treated with SDS, Triton X-100, and Tween-20, respectively (Figure 3a). Among the 3 detergents used and compared with the intact virus regarding the interaction with immuno-AuNPs, the maximum resonance change was observed for Triton X-100. Furthermore, non-ionic detergents (Triton X-100 and Tween-20) seemed to be more effective than the ionic detergent (SDS) (Figure 3b).

### Triton X-100 Titrations for A/Udorn/307/1972 Detection

**Figure 4:**
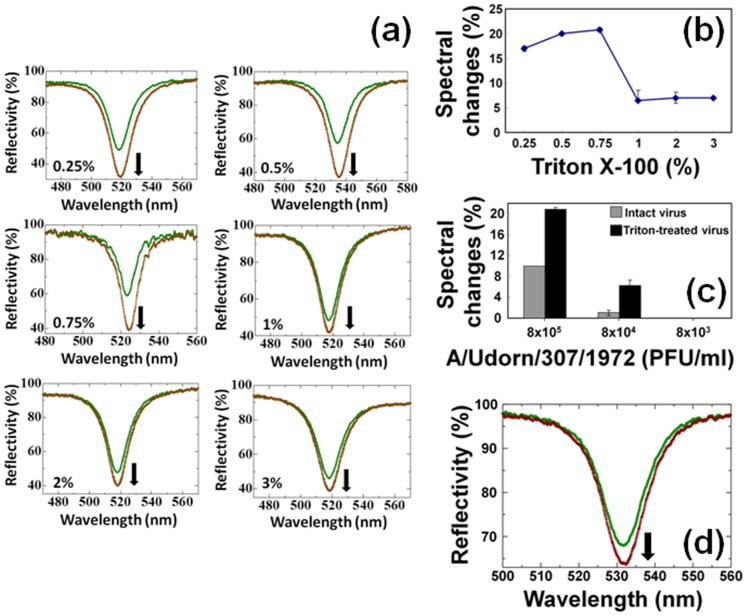
Analyses of Triton X-100 treated A/Udorn/307/1972 with immuno-AuNPs. (a) Spectrum shows the interactions of A/Udorn/307/1972 and immuno-AuNPs. The virus was treated with different titers of Triton X-100 (0.25, 0.5, 0.75, 1, 2, and 3%). Shift only occurs after the final. The green and brown lines represent the reflectivity measured after the attachment of virus and immuno-AuNPs, respectively, on the CDI surface immobilized with the capture antibody and ethanolamine. Vertical arrows on the figures indicate the direction of the spectral changes. (b) Graphical representation of titrated Triton X-100 with viruses. Error bars are shown with averaged values. (c) Analyses of the signal magnitude of the waveguide sensing system using intact and Triton X-100 treated A/Udorn/307/1972. Error bars are shown with averaged values. (d) Waveguide spectrum with Triton X-100 treated 8 ×10^4^ PFU/ml viruses. The green and brown lines represent the reflectivity measured after the attachment of the virus and immuno-AuNPs, respectively, on the CDI surface immobilized with the capture antibody and ethanolamine. Vertical arrow on the figures indicate the direction of the spectral changes.

We also treated the virus with different concentrations of Triton X-100. For this experiment, we pre-treated 8×10^5^ PFU/ml A/Udorn/307/1972 virus with 0.25%, 0.5%, 0.75%, 1%, 2%, and 3% Triton X-100 in the buffer solution used for the interactive analyses. Using these titers, the experiments were performed as described above, and the resulting spectral changes showed the values of 17±0.5, 20, 20.8±0.4, 6.5 (+2 or –0.3), 7±1.1, and 7±0.3, respectively (Figure 4a). From these results, it was predicted that with increasing concentrations of Triton X-100, signal magnitude would increase from 0.25% to 0.75%. When the Triton X-100 concentration was further increased to higher than 0.75% (from 1% to 3%), a sudden drop in signal reflectivity was observed. These changes may have been caused by the complete disintegration of viral proteins in the presence of extra Triton X-100 or the removal of the capturing antibody from the sensing plate. The linear changes with the different concentrations of Triton X-100 and the decrease after 1% concentration are indicated graphically (Figure 4b).

The above experiments confirmed that 0.75% Triton X-100 provides optimal release of proteins from the membrane of the A/Udorn/307/1972 virus; therefore, we treated viruses at different concentrations (8×10^5^, 8×10^4^, and 8×10^3 ^PFU/ml) with 0.75% Triton X-100 and monitored the signal magnitude of the waveguide-mode sensor. Good signal changes were observed with a viral concentration of 8×10^5 ^PFU/ml, which gave a reflectivity change of more than 20% (with the detergent-free treatment it was ∼10%), whereas when the viral concentration was reduced 10-fold to 8×10^4 ^PFU/ml, the reflectivity changes were reduced to approximately 6% (Figure 4c & d). Under these conditions, the signal magnitude increased in comparison to the non-detergent mix. Higher signal magnitude in the presence of lower concentrations of detergents (i.e., up to 0.75%) in comparison with the detergent-free situation suggests that other proteins released from viruses upon the disintegration of the viral membrane may also be detected. Because the antibody was raised against the whole virus particles, it is possible that the antibody also interact with influenza viral proteins other than HA. Western blot analyses confirmed this suspicion, as a prominent band was observed for membrane protein (M1), which originally resides inside the influenza virus (Figure 2c). In contrast, in the absence of detergents, the detected proteins were limited to the surface proteins of the influenza virus.

### Pre-mixed Immuno-AuNPs Form a Confined Ring of Nanodots on the Surface of A/Udorn/307/1972

**Figure 5:**
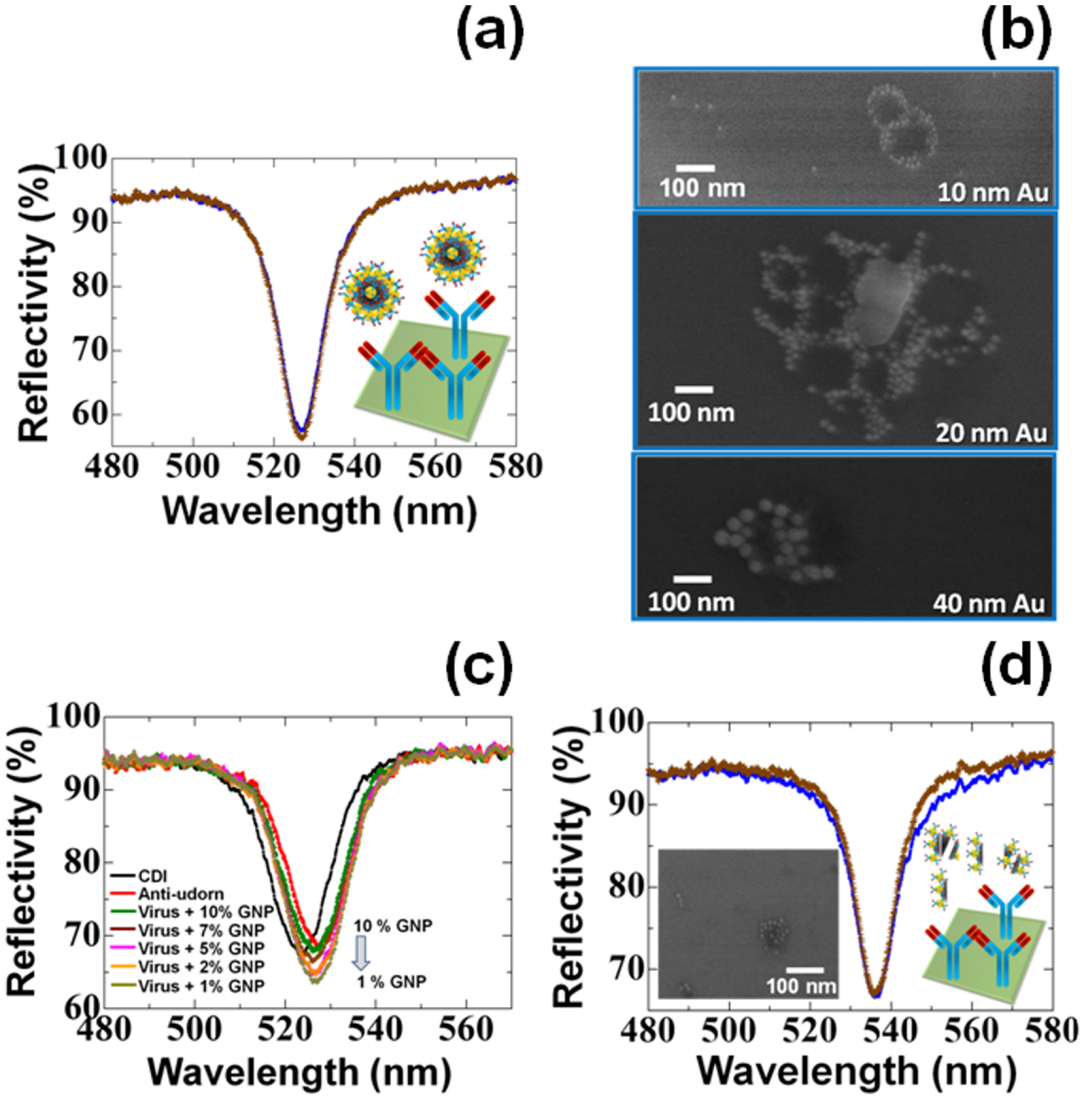
Analyses of pre-mixed A/Udorn/307/1972 with immuno-AuNPs. (a) Waveguide spectrum of 8×10^5^ PFU/ml viruses that were pre-mixed with immuno-AuNPs; 10% of the immuno-AuNPs were used. Vertical reflectivity changes are considered. Gold nanodots forming a confined ring on the surface of the virus are shown as figure inset. The blue and brown lines represent the reflectivity measured after the attachment of ethanolamine and immuno-AuNP-virus complex, respectively, on the CDI surface immobilized with anti-A/Udorn/307/1972 capture antibody. (b) SEM images shown with “O” ring formed on the viral surface. Different sizes of AuNP particles were used with diameters of 10, 20, and 40 nm. (c) Spectral analyses with the waveguide using different concentrations of immuno-AuNPs as the pre-mixer with the virus. immuno-AuNPs from 10% to 1% were used as the decreasing titer. (d) Waveguide spectrum with 8×10^5^ PFU/ml Triton X-100–treated viruses that were pre-mixed with immuno-AuNPs. Vertical reflectivity changes are considered. Formation of nanodots on the surface of the virus is shown as a figure inset. The blue and brown lines represent the reflectivity measured after the attachment of ethanolamine and immuno-AuNP-virus complex, respectively, to the CDI surface immobilized with anti-A/Udorn/307/1972 capture antibody.

To further increase the detection limit, we also pre-mixed A/Udorn/307/1972 virus with immuno-AuNPs and attached the resulting complexes to the sensing surface immobilized with the capture antibody. When we performed this experiment, the complete loss of spectral changes, i.e., lack of detection of the virus, was surprising (Figure 5a). To directly visualize these dramatic changes, we observed the CDI-functionalized surface attached with pre-mixed immuno-AuNP complexes and viruses under SEM. To make these attachments stable, steps with immobilizing capture antibody and extensive washing was avoided. The SEM images showed that AuNPs were arranged on the membrane of the virus and formed a ring-shaped structure (Figure 5b). Based on this result, we considered that the formation of confined gold nanodots on regions of the viral surface did not leave space for attachment of the capture antibody, and that the immuno-AuNP virus complexes thus ultimately remained in the buffer solution and were subsequently washed out during the washing steps in waveguide measurements (Figure 5a; inset). To confirm the nanodot arrangement on the viral surface, we also conducted experiments with 20- and 40-nm AuNPs conjugated with antibodies. These experiments also gave a similar pattern of nanodots that formed an “O” ring on the viral surface with the expected sizes under SEM observation (Figure 5b). We therefore further tested different dilutions of immuno-AuNP pre-mixed with the viruses by waveguide, as we expected some binding of these complexes to the capture antibody immobilized on the sensing plate. We prepared serial dilutions of pre-mixed virus-immuno-AuNP complexes from 10% to 1%. As expected, apparent spectral changes were not observed with 10% immuno-AuNP as described above, whereas with decreasing amounts of immuno-AuNP, some attachment occurred. It seems that a lower concentration of immuno-AuNP may not completely cover the surface of the virus, thus leaving some space and attached with capture antibody (Figure 5c). Similar to the pre-mixing of the intact virus and immuno-AuNP, we also prepared a pre-mix in the presence of 0.75% Triton X-100 and observed it using the waveguide sensing plate. This mix gave results similar to those of the experiments in the absence of detergent, and no spectral changes were observed. (Figure 5d). The sensing plate with the pre-mix attached was also visualized under SEM, which showed the formation of gold nanodots on the viral particles, but without the formation of a ring structure, as the viruses are fragmented with detergent treatment (Figure 5d; inset). However, the binding of immune-AuNP on the fragmented viruses was clear.

**Figure 6:**
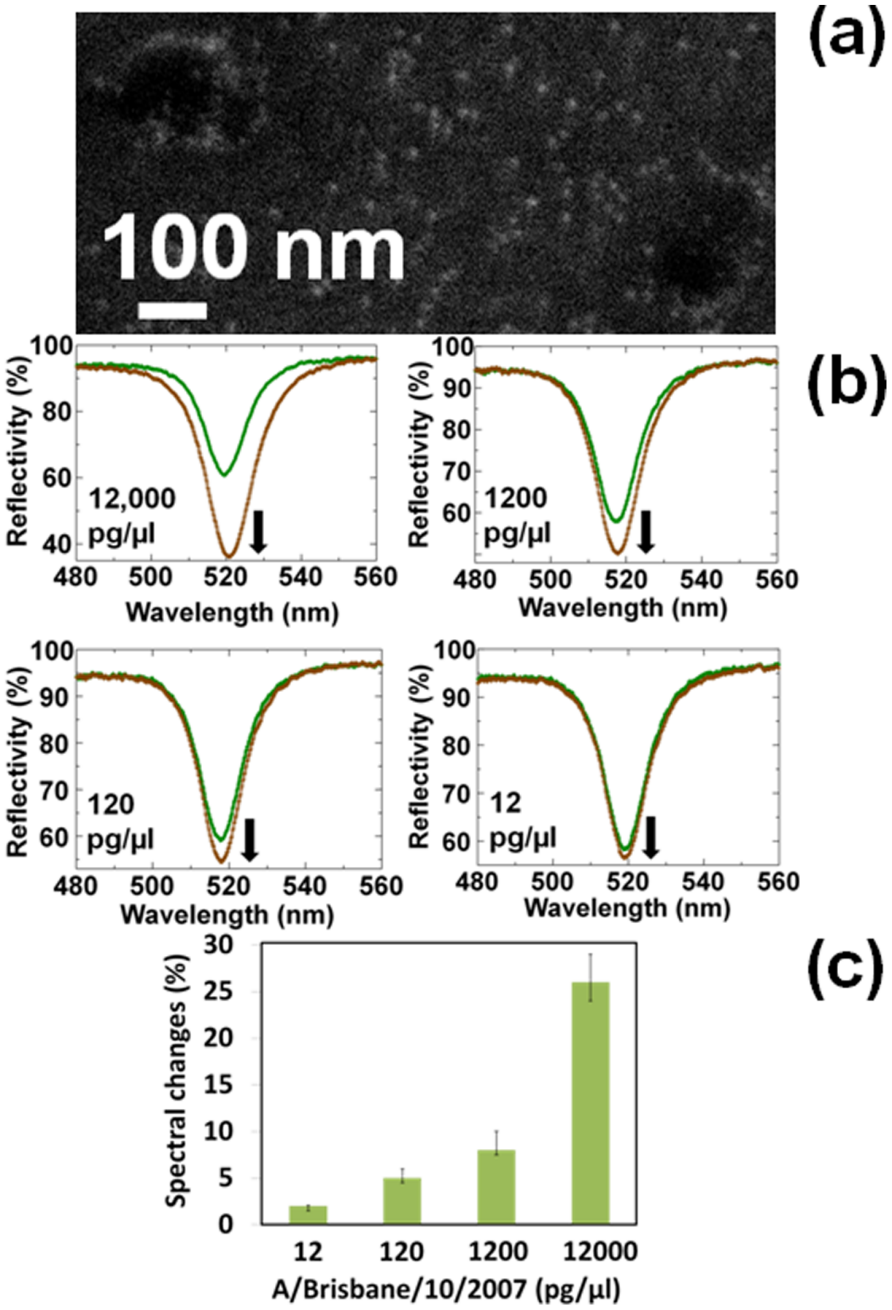
Interactions of A/Brisbane/10/2007 and immuno-AuNP. (a) SEM images shown with nanodots formed on the ruptured A/Brisbane/10/2007 surface. Size of immuno-AuNP particles used with diameter of 10 nm. (b) Spectrum shows the interactions of A/Brisbane/10/2007 and immuno-AuNPs. Different dilutions of viruses were used (1∶100, 1∶1000, 1∶10000 and 1∶100,000). These dilutions represent 12,000, 1,200, 120, and 12 pg/µl, respectively. Shift only occurs after the final. The green and brown lines represent the reflectivity measured after the attachment of virus and immuno-AuNPs, respectively, on the CDI surface immobilized with the capture antibody and ethanolamine. Vertical arrows on the figures indicate the direction of the spectral changes. (c) Graphical representation of spectral changes with the interactions of A/Brisbane/10/2007 and immuno-AuNPs. Error bars are shown with averaged values. Values less than 3% was considered to be within the error ranges and the minimum detectable limit is 120 pg/µl of A/Brisbane/10/2007.

Similar to the above studies with waveguide measurements, we also keen to test other H3N2 strain, A/Brisbane/10/2007 which is commercially available. The origin of this strain is very far from A/Udorn/307/1972 and the possibility of interaction of HA from A/Brisbane/10/2007 and immuno-AuNP is lesser. However, proteins other than HA from A/Brisbane/10/2007 may react well with the anti-A/Udorn/307/1972. To explore this possibility, we tried to observe the formation of ring structure on A/Brisbane/10/2007 under SEM. The observed SEM images have a clear gold nanodots, however, they were on the ruptured viral particles and not forming any ring structure (Figure 6a). These images clearly declared that analyses with the availed A/Brisbane/10/2007 would interact other proteins reside in the virus or HA2 to which immuno-AuNP could not access on intact virus particles by some steric constraint. To confirm the interactions of these proteins, we tested on the capturing antibody immobilized waveguide sensor surface. For the initial experiments we diluted the supplied stock virus (1.2 µg/µl) by 100-fold, thus giving a final concentration of 12 ng/µl. Spectral measurements with the vertical changes for this interaction gave a reflectivity value of 26%. Further measurements were obtained at concentrations of 1200, 120, and 12 pg/µl. With these dilutions, the spectral changes measured using the waveguide sensor were 8, 5, and 2%, respectively (Figures 6b). At the lowest dilution (12 pg/µl), the detection limit was within the error range (less than 3%). However, with a 10-fold higher concentration (120 pg/µl), the spectral change was increased by 5%, indicates the detection level. In the case of A/Brisbane/10/2007, it seems other proteins such as M1 may react well with the anti-udorn antibody.

**Figure 7:**
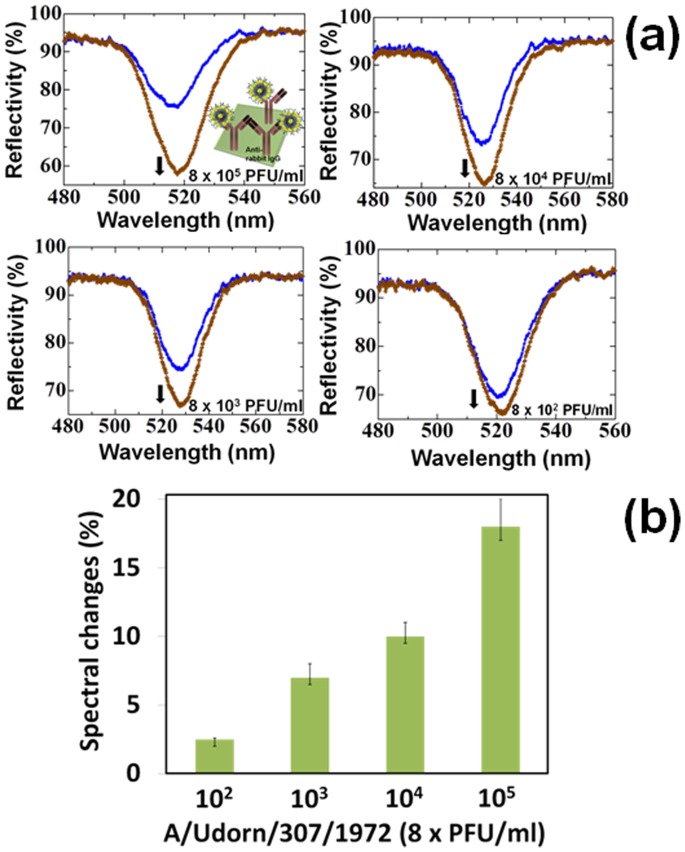
Analyses of pre-mixed A/Udorn/307/1972 with immuno-AuNPs on captured anti-rabbit IgG. (a) Waveguide spectrum of A/Udorn/307/1972 that was pre-mixed with immuno-AuNPs; Viruses from 8×10^2^ PFU/ml to 8×10^5^ PFU/ml were titrated. 10% of the immuno-AuNPs were used. Vertical reflectivity changes are considered. Gold nanodots forming a confined ring on the surface of the virus are shown as figure inset. The blue and brown lines represent the reflectivity measured after the attachment of ethanolamine and immuno-AuNP-virus complex, respectively, on the CDI surface immobilized with anti-rabbit IgG as the capture antibody. (b) Graphical representation of spectral changes with the above interactions. Error bars are shown with averaged values. Values less than 3% was considered to be within the error ranges and the minimum detectable limit is 8×10^3^ PFU/ml of A/Udorn/307/1972.

To make sure with the formation of the above complex, which forms a ringlike structure on the membrane of intact virus (A/Udorn/307/1972), the virus and immuno-AuNP complex was attached on the waveguide-sensing plate pre-immobilized with anti-rabbit IgG, which can recognize the immuno-AuNPs (anti-A/Udorn/307/1972 rabbit antibody on AuNPs). As the consequence of this reaction, we could see the clear spectral changes by the waveguide-mode sensor (Figure 7; inset), which confirms the genuine interaction between immuno-AuNPs and anti-rabbit IgG. Further, these complexes improved the spectral change and detected the viruses of 8×10^3^ PFU/ml. The dilution made beyond this concentration showed the spectral changes equal to the error range.

In the present investigation, immuno-AuNP was prepared to detect viruses belonging to H3N2 (A/Udorn/307/1972 and A/Brisbane/10/2007). Furthermore, the detection level of the assay was improved (from 8×10^5^ to 8×10^4 ^PFU/ml) by using detergents, especially Triton X-100 with a concentration of less than 0.75%. In an another approach, immuno-AuNP complexes formed confined nanodots on the surface of both intact and detergent-treated viruses and were easily visualized by microscopy. However, nanodot ring formation did not show any spectral changes on the waveguide sensor as the complex prevented the binding between capture antibody and immune-AuNPs, and this structure could be visualized on the CDI surface by SEM analyses. However, these complexes improved the detection limit to 8×10^3^ PFU/ml when captured on the pre-immobilized sensing plate with anti-rabbit IgG. These studies introduce the possibility of observing trapped intact viruses on sensing plates that utilize optical sensing systems.

## Materials and Methods

### Reagents and Biomolecules

We purchased *N,N*′-carbonyldiimidazole (CDI) and Gradient polyacrylamide pre-cast gels from Wako Chemicals (Osaka, Japan). A/Brisbane/10/2007 virus belonging H3N2 was from Prospec (Rehovot, Israel). Horse radish peroxidase-conjugated anti-rabbit immunoglobulin G was purchased from Promega, Medison USA. Clear Blot membrane-P and nitrocellulose membranes were purchased from ATTO (Tokyo, Japan) and Millipore (MA, USA), respectively. Triton, Tween-20, and sodium dodecyl sulfate were from Wako Chemicals (Wako, Japan). The enhanced chemiluminescence kit was from Amersham Pharmacia Biotech (PA, USA). All samples were stored according to the suppliers’ recommendations.

A/Udorn/307/1972 virus was propagated in MDCK cells in minimum essential medium (MEM; GIBCO, Gland Island, NY, USA) containing 2.5 µg/ml TPCK-trypsin (Worthington Biochemical Corporation, Lakewood, NJ, USA) and penicillin and streptomycin (100 units/ml each, GIBCO, Gland Island, NY, USA) antibiotics at 34°C in 5% CO_2_ for virus preparation with cleaved HA and without TPCK-trypsin for virus preparation with uncleaved HA. The culture fluid was harvested 24 hr after infection with a multiplicity of infection of 1.0 and virions were purified by one cycle of differential centrifugation and two cycles of sucrose gradient centrifugation, as described previously [39].

### Preparation of EFC-WM Sensor Chips and Measurements

The EFC-WM sensor uses a sensing plate with a multilayer structure that consists of a dielectric waveguide, higher refractive index layer, and glass substrate [30]. The sensing plate illuminated under the Kretschmann configuration operates as a sensor that is capable of detecting modifications in the dielectric environment near the waveguide surface by measuring changes in reflectivity [40]. In the present study, we utilized a compact optical system of surface plasmon resonance sensors as a spectral readout system in conjunction with the EFC-WM sensor [26]. The optical setup is shown in Figure 1. In this system, a Xe lamp was used as a light source. The light from the lamp was guided to a collimator lens; the collimated light was radiated into a prism where the incident angle was parallel to the bottom face of the prism. Then, the monolithic sensing plate placed on the bottom of a prism was illuminated, and the spectrum of the reflected light was recorded by a spectrophotometer. The prism was made of SiO_2_ glass, and the bottom angle of the prism was 38°C. A monolithic sensing plate that we developed previously was applied [30]. The monolithic sensing plate consisted of a SiO_2_ glass substrate, single crystalline Si layer, and thermally grown SiO_2_ waveguide. In the present experiment, the thickness of the SiO_2_ glass waveguide and single crystalline Si layer was 45 and 360 nm, respectively. The sensing system was adjusted to show a dip in reflectance at approximately 520 nm, which corresponds to the optical absorption of immuno-AuNPs. If AuNPs are attached to the waveguide surface, the dip will be deepened by the optical absorption of the AuNP [31].

### Detection of Immuno-AuNPs and Virus Conjugates on the Sensor Surface

The chips were treated with an alkaline solution for 30 min, washed thoroughly with water, and dried. Modification with 0.5 M CDI in dioxane on the alkaline-treated sensor chip was performed, followed by air drying. The reaction was conducted at 37°C for 2 h, followed by rinsing with acetone then water. On this surface, the capture antibody (500 nM) was immobilized and blocked by 1 M ethanolamine. Virus samples were placed on this antibody-immobilized sensor surface, and further signal measurements were obtained by attaching immuno-AuNPs. The coupling reaction for CDI and the antibody was conducted for 3 h; other incubations were performed for 30 min at room temperature.

### Direct Visualization of Pre-mixed Immuno-AuNPs and Viruses

For the direct visualization of immuno-AuNPs bound to the viruses, the CDI modifications and the attachment of the capture antibody on the sensor chips were conducted as described above. Next, immuno-AuNPs and intact viruses (8×10^5^ PFU/ml) were pre-mixed and attached to the pre-immobilized antibody surface after incubation at room temperature for 10 min to allow immuno-AuNPs to bind to the viruses. Measurements were obtained by the waveguide sensor system after incubation of the samples on the sensing plate for 30 min. The chip surface was also visualized by scanning electron microscopy (SEM) (JEOL JSM-6335 F) after the complex was directly immobilized on the CDI surface. For SEM analyses with pre-mixed samples, no capturing antibody were used. To determine the specificity of these complexes, the CDI-modified sensing plate was immobilized with anti-rabbit IgG and interacted with these complexes.

### Western Blot Analyses

The specificity of the antibody for the viruses was analyzed by standard Western blotting with an anti-Udorn antibody. In brief, viral proteins were separated by SDS-polyacrylamide gel electrophoresis as described before [41] and transferred to polyvinylidene difluoride membrane. After immobilization of the protein on the membrane, the membrane was blocked with 3% nonfat milk in TBS buffer at room temperature for 1 h and then treated with a 1∶10,000 dilution of the primary antibody (anti-Udorn antibody) at 4°C overnight. After the unbound primary antibody was removed by washing, the membrane was incubated for 1 h with horseradish peroxidase –conjugated goat anti-rabbit immunoglobulin G. The immunocomplexes thus formed were visualized through staining by using a substrate for horseradish peroxidase. The detection of antigen–antibody interactions was visualized by using an enhanced chemiluminescence kit.

## Supporting Information

Figure S1 **Spectrum shows the non-specific attachment of AuNP-antibody conjugates.** AuNP diameters with10, 20 and 40 nm are shown. The black, red and blue lines represent the reflectivity measured after the attachment of CDI, antibody and ethanolamine, respectively. No viruses were used. In 10 nm AuNP attachments, green, brown, pink, violet and orange lines represent dilutions of 1∶200, 1∶100, 1∶50, 1∶20 and 1∶10 (AuNP:PBS), respectively. In 20 nm AuNP attachments, green, brown and pink lines represent dilutions of 1∶200, 1∶100 and 1∶50 (AuNP:PBS), respectively. In 40 nm AuNP attachments, green, brown, pink and violet lines represent dilutions of 1∶200, 1∶100, 1∶50 and 1∶20 (AuNP:PBS), respectively.
(TIF)

Figure S2 **Spectrum shows the interaction of AuNP-antibody conjugates with pre-immune serum**. The green and brown lines represent the reflectivity measured after the attachment of virus and IAuNP, respectively. Pre-immune serum collected from the same rabbit used for immunization. Similar concentration (500 nM) of serum was used for capturing as in the specific experiments.
(TIF)
