# Correction: Observations of Immuno-Gold Conjugates on Influenza Viruses Using Waveguide-Mode Sensors

**DOI:** 10.1371/annotation/7bb0ff7b-3527-4a42-a50a-ec81f108ac41

**Published:** 2013-12-20

**Authors:** Subash C. B. Gopinath, Koichi Awazu, Makoto Fujimaki, Kazufumi Shimizu, Takayuki Shima

There is an error in the last sentence in the Figure 1 legend and the third paragraph of the Material and Methods section. Xe lamp is incorrect. The correct sentence for both should read: A halogen lamp was used as the light source.

The word “step” was omitted from the sentence, “Shift only occurs after the final” in the legends for Figure 3, 4, and 6. The corrected sentences should read, “Shift only occurs after the final step.”

There is an error in the title of reference 31. The correct reference is: Fujimaki M, Nomura K, Sato K, Kato T, Gopinath SCB, et al. (2010) Detection of colored nanomaterials using evanescent field-based waveguide sensors. Opt Exp 18: 15732-15740.

