# Supplementary figures and images for: Observations of Immuno-Gold Conjugates on Influenza Viruses Using Waveguide-Mode Sensors
Source: PLoS One. 2013 Jul 11;8(7):e69121. doi: 10.1371/journal.pone.0069121 (PMC3708897; doi:10.1371/journal.pone.0069121)

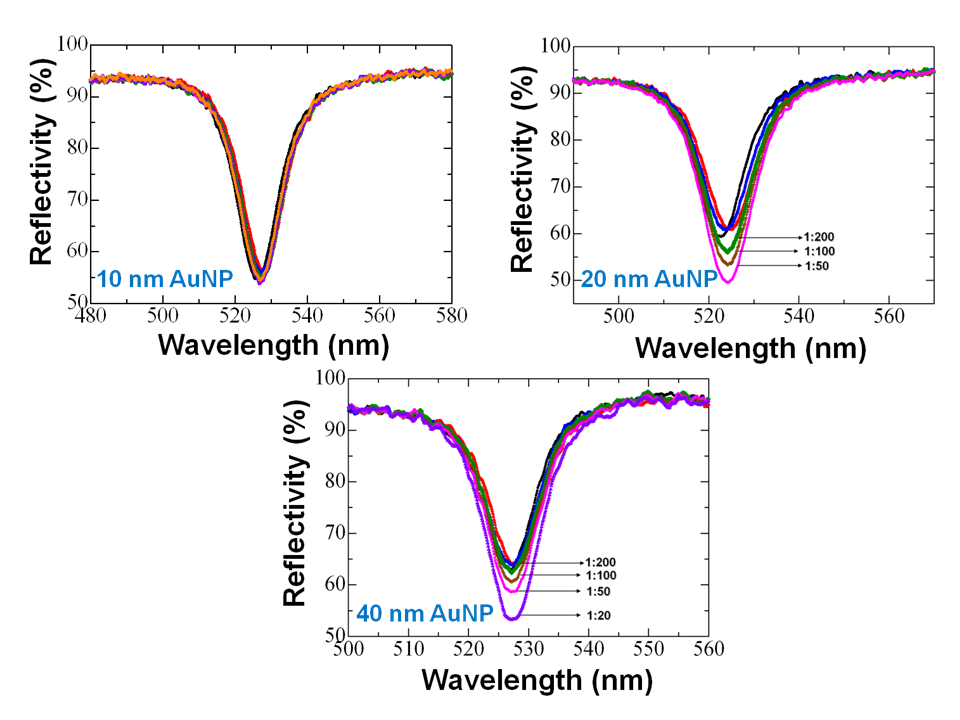

Supplement: Figure S1 — Spectrum shows the non-specific attachment of AuNP-antibody conjugates. AuNP diameters with10, 20 and 40 nm are shown. The black, red and blue lines represent the reflectivity measured after the attachment of CDI, antibody and ethanolamine, respectively. No viruses were used. In 10 nm AuNP attachments, green, brown, pink, violet and orange lines represent dilutions of 1∶200, 1∶100, 1∶50, 1∶20 and 1∶10 (AuNP:PBS), respectively. In 20 nm AuNP attachments, green, brown and pink lines represent dilutions of 1∶200, 1∶100 and 1∶50 (AuNP:PBS), respectively. In 40 nm AuNP attachments, green, brown, pink and violet lines represent dilutions of 1∶200, 1∶100, 1∶50 and 1∶20 (AuNP:PBS), respectively. (TIF) [file pone.0069121.s001.tif]

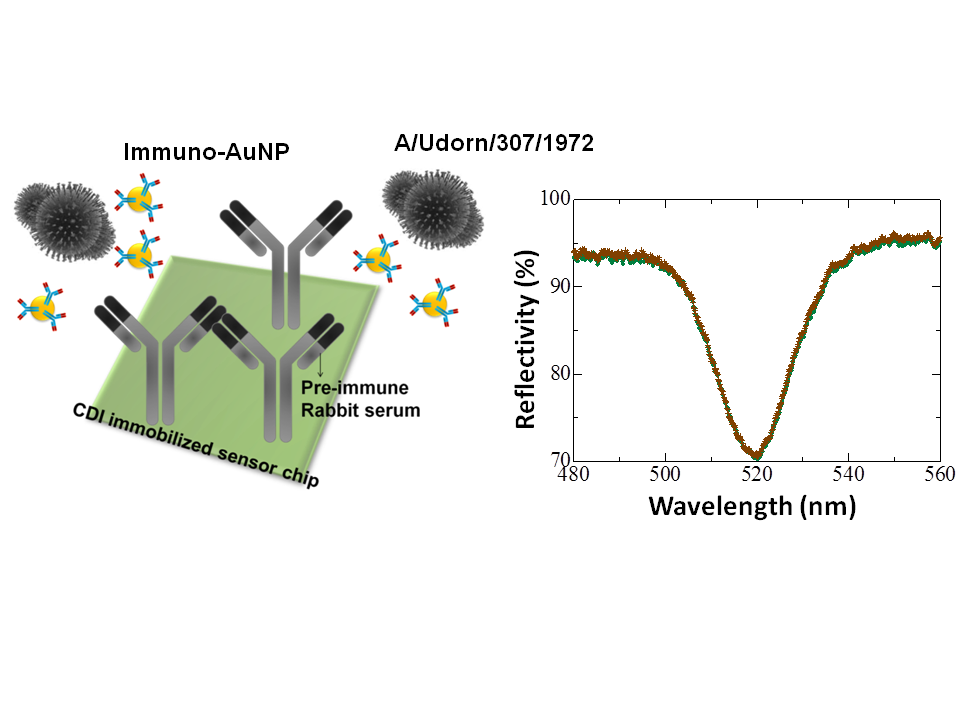

Supplement: Figure S2 — Spectrum shows the interaction of AuNP-antibody conjugates with pre-immune serum. The green and brown lines represent the reflectivity measured after the attachment of virus and IAuNP, respectively. Pre-immune serum collected from the same rabbit used for immunization. Similar concentration (500 nM) of serum was used for capturing as in the specific experiments. (TIF) [file pone.0069121.s002.tif]
